# The Age-Specific Cumulative Incidence of Infection with Pandemic Influenza H1N1 2009 Was Similar in Various Countries Prior to Vaccination

**DOI:** 10.1371/journal.pone.0021828

**Published:** 2011-08-05

**Authors:** Heath Kelly, Heidi A. Peck, Karen L. Laurie, Peng Wu, Hiroshi Nishiura, Benjamin J. Cowling

**Affiliations:** 1 Victorian Infectious Diseases Reference Laboratory (VIDRL), North Melbourne, Victoria, Australia; 2 WHO Collaborating Centre for Reference and Research on Influenza, Victorian Infectious Diseases Reference Laboratory (VIDRL), North Melbourne, Victoria, Australia; 3 School of Public Health, The University of Hong Kong, Hong Kong Special Administrative Region, China; 4 PRESTO, Japan Science and Technology Agency, Saitama, Japan; University of Sao Paulo, Brazil

## Abstract

**Background:**

During the influenza pandemic of 2009 estimates of symptomatic and asymptomatic infection were needed to guide vaccination policies and inform other control measures. Serological studies are the most reliable way to measure influenza infection independent of symptoms. We reviewed all published serological studies that estimated the cumulative incidence of infection with pandemic influenza H1N1 2009 prior to the initiation of population-based vaccination against the pandemic strain.

**Methodology and Principal Findings:**

We searched for studies that estimated the cumulative incidence of pandemic influenza infection in the wider community. We excluded studies that did not include both pre- and post-pandemic serological sampling and studies that included response to vaccination. We identified 47 potentially eligible studies and included 12 of them in the review. Where there had been a significant first wave, the cumulative incidence of pandemic influenza infection was reported in the range 16%–28% in pre-school aged children, 34%–43% in school aged children and 12%–15% in young adults. Only 2%–3% of older adults were infected. The proportion of the entire population infected ranged from 11%–18%. We re-estimated the cumulative incidence to account for the small proportion of infections that may not have been detected by serology, and performed direct age-standardisation to the study population. For those countries where it could be calculated, this suggested a population cumulative incidence in the range 11%–21%.

**Conclusions and Significance:**

Around the world, the cumulative incidence of infection (which is higher than the cumulative incidence of clinical disease) was below that anticipated prior to the pandemic. Serological studies need to be routine in order to be sufficiently timely to provide support for decisions about vaccination.

## Introduction

A novel pandemic influenza virus, influenza A H1N1 2009 (pH1N1), was identified in North America in April 2009 and rapidly spread around the world [1]. Urgent priorities at the start of the pandemic were to determine the transmissibility of the new virus and the severity of resulting infections, in order to judge the appropriate scale of the pandemic response. When anticipating the arrival of pandemic specific vaccines later in 2009, a key public health priority at that time was to identify the optimal target groups for vaccination. This depended on the course of the pandemic, specifically on the proportion of children that had been infected, as well as the groups in the population considered to be most at risk of an adverse outcome.

An early serological study in the US suggested that most of the population would likely be susceptible to infection with the new virus, but that older people may have had some protection against pH1N1 [2]. Consistent with this suggestion were the early epidemiologic observations of higher clinical attack rates in children [3]. However influenza virus infections can often be subclinical, and there may be variation in subclinical infections by age [4]. In order to fully understand the impact of the 2009 pandemic, and to be able to make comparisons with previous pandemics and seasonal influenza viruses, estimates of the cumulative incidence of pH1N1 infection were needed.

Various terms have been used to describe measures of infection. The ‘attack rate’ describes the cumulative incidence of infection during a defined disease outbreak [5], although attack rate is sometimes used to refer only to clinical cases. While ‘rate’ is generally associated with a measurement of time in the denominator, ‘attack rate’ does not include time. Similarly the ‘infection rate’ in a specified time refers to the cumulative incidence of infection, both symptomatic and asymptomatic. In this review we focus on serological studies that were conducted to estimate the cumulative incidence of symptomatic and asymptomatic infection.

Early in the course of the pandemic, a number of approaches were used to estimate the cumulative incidence of pH1N1 infection. Studies that extrapolated from surveillance data and surveys required assumptions about the proportion of infected people who were likely to seek medical care and the proportion of infections that were asymptomatic [6], [7]. However the most reliable method of estimating the cumulative incidence of infection, including asymptomatic infections, is serological testing, given that most infected individuals will develop humoral antibody at detectable titres 2–3 weeks after infection [4].

Two alternative serological study designs were used to infer the cumulative incidence of infection with pH1N1. In a longitudinal design, sera were collected from individuals before and after a period of pH1N1 circulation, and infected individuals were identified by comparison of paired antibody titres. Individuals were typically classified as infected if there was a 4-fold or greater rise in antibody titre across the paired sera. In a serial cross-sectional design, the prevalence of antibody at a certain threshold (seropositivity) was compared before and after a period of pH1N1 circulation. Alternative thresholds were used for comparisons of seroprevalence, for example 32 [4], 40 [8], [9], [10] or 10 [11]. Estimates of the cumulative incidence of infection varied, depending, amongst other things, on the extent of pandemic spread prior to the availability of vaccination, the sensitivity and specificity of the tests used and the cut-off titre used to define infection.

We review serosurveys which estimated the cumulative incidence of pH1N1 in the general community and were published between 14 April 2009, when the novel pH1N1 virus was first identified, and 22 December 2010, which allowed more than a year since the end of the first pandemic wave in the northern hemisphere and the end of the season in which pH1N1 predominated in the southern hemisphere, and before a second season in which pH1N1 circulated. We aimed to compare study designs and age-specific estimates of the cumulative incidence of infection with pH1N1 in unvaccinated populations.

## Methods

To identify published studies two of the authors (HP, BJC) designed a search of the database PubMed. One author (HP) performed an initial search on 22 December 2010 and two authors (KL, HP) repeated the search on 27 April 2011, using the PubMedAdvanced Search engine. Articles were searched in ‘All fields’ using the following terms:

‘influenza’ or 'flu' or ‘influenza A’ or 'H1N1' or 'pH1N1' or 'A/H1N1' or 'A(H1N1)' or 'pdmH1N1' or 'H1N1pdm' or 'H1N1swl' or 'H1N1soiv' or '2009(H1N1)' or '(H1N1)2009'
‘infect*’ or ‘antibod*’ or ‘immun*’ or ‘protect* or ‘prevalence’ or ‘attack rate’ or ‘incidence’ or ‘sero*'
(1) and (2).

We limited articles to those published in English between 14 April 2009 and 22 December 2010 (epub dates). The title of each article identified by the searches was reviewed for relevance. Abstracts were then reviewed for eligibility based on a broad description of serological assessment of pH1N1 infection in a general community setting. In addition we followed up references in each of the eligible studies and sought information on other serological studies from expert colleagues in various disciplines. We also performed regular searches of journals (*Eurosurveillance*, *PLoS One and PLoS Currents Influenza*) which were known to publish early pandemic influenza studies, including serosurveys. A further search in Google Scholar using the search terms ‘influenza’ AND ‘2009’ AND ‘antibody’ did not indentify any other studies.

Three of the authors (HK, HP, KL) assessed the eligibility of studies for inclusion and performed the data extraction. To be included a study needed to report estimates of the cumulative incidence of pH1N1 infection from a cross-sectional or longitudinal community-based study. We used conventional criteria for the definition of pH1N1 infection. In a longitudinal study a four-fold rise in microneutralisation (MN) or haemagglutination inhibition (HI) titres (to ≥40) in paired samples was considered evidence of infection. In the cross-sectional design titres ≥32 in a single HI assay or ≥40 in a single MN assay were considered to be evidence of infection. We excluded studies that provided only estimates of infection after the circulation of pH1N1, with no pre-pandemic sample, or studies that provided only estimates of the prevalence of antibodies that cross-reacted to pH1N1 before the pandemic. We also excluded studies which were unable to distinguish antibody response due to natural infection from those due to vaccination, since vaccination would have altered the risk of infection with pH1N1. Disagreement among authors was resolved by discussion until a consensus was reached. Data extracted from the studies included the age-specific estimate of the cumulative incidence of infection, with confidence intervals if reported, the timing of serological sampling in relation to the circulation of pH1N1 and the serological assay(s) used. The months of pandemic circulation were obtained from surveillance data in the public domain when not stated in the studies included in the review.

We used the difference in proportions of individuals in specified age groups with antibody titre >1∶40 before and after the first pandemic wave to estimate the cumulative incidence of infection in the studies included. However since only around 90% of convalescent individuals had antibody titre >1∶40 [4], [12] we inflated the observed cumulative incidence of infection by 10% when estimating the true cumulative incidence of infection. We estimated the direct age-standardization of the cumulative incidence of infection using the age-specific population data for each country included in the final review. We used official government data for 2009, except for India, where the most recent age-specific population data was extracted from the 2001 Census [13], [14], [15], [16], [17], [18], [19].

Since influenza spreads from person to person and the risk of infection for an individual is not independent from the risk of infection for others in the population, confidence intervals based on an independence assumption may underestimate the uncertainty associated with estimates of the cumulative incidence of infection from serologic studies [20]. We used an approach which accounts for this non-independence to re-estimate confidence intervals for the estimates of the cumulative incidence of infection. The variance of the cumulative incidence of infection was estimated using the equation below:
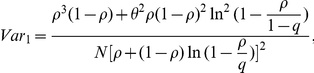
where 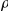 is the estimated mean age-standardized cumulative incidence of infection, 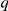 is the proportion of individuals with pre-existing immunity, and 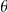 refers to the coefficient of variation of the generation time of influenza. 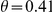 was applied in the calculation based on the contact tracing data collected in the Netherlands [20]. *N* represents the sample size of the population of interest. We also considered the uncertainty introduced by the diagnostic method in the study by applying the equation to estimate the variance [21]:
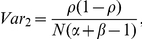
where α and β denote the sensitivity and specificity of the diagnostic method of interest, which were assumed to be 0.9 and 1.0, respectively, in our analyses. The variance for the estimated CII was then given by *Var_1_ + Var_2_*. The re-calculated confidence intervals assumed simple random sampling.

We report the original results from the studies and compare these with our revised estimates of the cumulative incidence of infection with re-calculated confidence intervals.

## Results

### Studies identified

**Figure 1:**
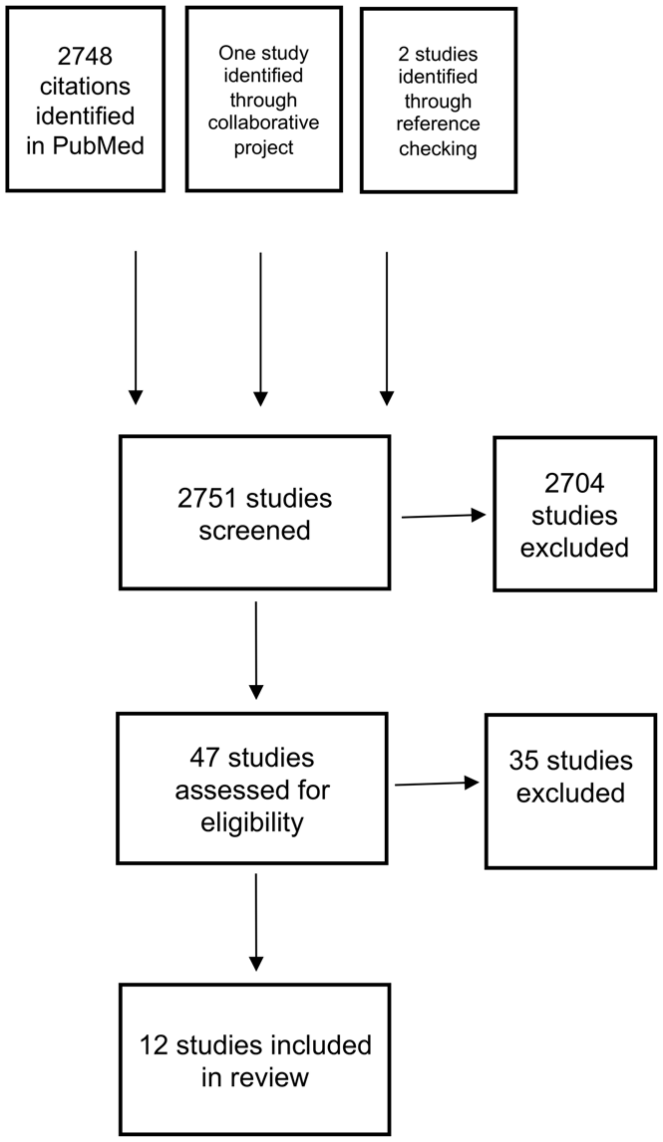
Flowchart for the review process.

The search identified 2748 citations. One study was identified through a collaborative project [8] and two studies were identified through reference checking [22], [23]. We identified 47 relevant published studies (Figure 1). A total of 35 studies were excluded. Four were commentaries and letters regarding pH1N1 serological studies [24], [25], [26], [27], two studies were duplicates [28], [29], and 29 studies did not match our inclusion criteria. These studies were conducted in Canada [30], China [31], [32], [33], [34], [35], [36], England [37], Finland [38], [39], France [22], Germany [40], Hong Kong [12], India [11], Iran [41], Italy [42], Japan [23], Scotland [43], Singapore [44], [45], [46], [47], [48], Taiwan [49], [50], [51], the United States [2], [52], and a multi-location study that included the United States, Costa Rica, Europe and Japan [53]. We identified one further study on pre-existing pH1N1 immunity in the general population that examined only the molecular basis for immunity [54]. This study was not considered further in the review. The specific reasons for exclusion of all studies are outlined in Table S1.

### Studies included in the review

Twelve studies were included in the review (Table 1). These studies were conducted in Australia [8], [9], [55], [56], Canada [57], England [4], Hong Kong [10], [58], India [59], New Zealand [60], Norway [61], and Singapore [62]. Not all studies included all age groups, with the absence of children being the most common omission (Table 1).

**Table 1:** Outline of studies included in the review.

Study location	pH1N1 circulation	Sampling time points		Summary of study design	Comments a
		Pre pH1N1	Post pH1N1		
Australia [55]	May - Sep 2009	April - early May 2009	Oct-Dec 2009	Random plasma samples of blood donors. Age stratified.	Pre-sample (n = 501) from two cities; post-sample (n = 1307) from eight cities, including the site that provided the pre-sample.
New South Wales, Australia [9]	June -Sep 2009	2007-2008	Aug-Sep 2009	Pre: serological samples submitted for non-influenza testing. Post: residual diagnostic plasma and sera.	Pre-sample (n = 474), post-sample (n = 1247). Sampling strategy was different for pre and post samples but both were convenience samples of residual diagnostic sera.
Victoria, Australia [56]	May-Aug 2009	2008-early 2009	Aug-Oct 2009	Pre: archive sera from healthy adults. Post: opportunistic sample from existing cohort, which used a stratified cluster sample and sequential serological sampling.	Pre-sample n = 100. Post-sample n = 706 represented a 34% response rate.
Western Australia, Australia [8]	June-Sep 2009	Nov 2008-May 2009	Aug-Nov 2009	Convenience samples of residual diagnostic sera. Samples collected for respiratory infections excluded. Only children and pregnant women sampled.	Pre- and post-sampling strategies identical and approximately balanced (n∼450 children and ∼200-300 pregnant women). Post sampling at two time points; not all samples were post-pandemic.
Manitoba, Canada [57]	April-July 2009	March 2009	Aug 2009	Random samples of frozen stored sera from pregnant women tested as part of routine pre-natal care	Pre- (n = 252) and post- (n = 296) samples were approximately balanced and selected by the same sampling strategy.
England [4]	June-Aug 2009 (1^st^ wave)	2008-April 2009	Aug-Sep 2009	Convenience sera from national sero-epidemiology programme and residual samples from biochemistry testing.	Pre- (n = 1403) and post- (n = 1954) samples were approximately balanced and selected by the same sampling strategy.
Hong Kong [10]	Aug-Oct 2009	Before Aug 2009	After 15 Nov 2009	Convenience samples of blood donors, hospital outpatients and an existing paediatric cohort.	Blood donors n = 12,217. Hospital outpatients n = 2,520. Paediatric cohort pre-sample n = 151, post-sample n = 766. The paediatric cohort was part of a vaccine trial. Samples were tested by MN assay.
Hong Kong [58]	Aug-Oct 2009	2008	Nov - Dec 2009	Pre: residual virological diagnostic serum samples (1 to >65 years). Post: residual routine hepatitis B serum samples ( >9 years).	Pre-sample (n = 234), post-sample (n = 178). Small unbalanced sampling strategy with no post-pandemic samples for children under 9 years old.
Pune, India [59]	June-Aug 2009	Jan 2005-Mar 2009	Aug-Dec 2009	Pre: stored residual sera for dengue serology. Post: convenience samples of school children, school and medical staff, railway commuters, slum dwellers and the ‘general population’.	Pre-samples (n = 222) yielded low seropositivity (1%). Post-sampling strategy (n = 9233) not fully detailed and included repeat sampling in some groups. The ‘general population’ survey employed a cluster sample of households in 20 localities.
New Zealand [60]	April-Sep 2009	Before 22 April 2009	Nov 2009-March 2010	Pre: residual diagnostic sera from two laboratories. Post: targeted stratified random sample of patients registered at sentinel general practices.	Pre-sample n = 521. Post-sample n = 1156. Sentinel practices were involved in surveillance of influenza-like illness. A health care worker survey was not included in this review.
Norway [61]	July-Aug 2009; Oct –Nov 2009	August 2008	Aug 2009 and Jan 2010	Three age and geographically representative residual serum panels: pre-pandemic August 2008 (n = 689), post-pandemic panel August 2009 (n = 2116), post-national vaccination campaign January 2010 (n = 541).	August 2009 post-pandemic sample indicated minimal pH1N1 antibodies, attributed to small first wave in Norway. Unable to distinguish between antibodies from natural infection or vaccination in January sample.
Singapore [62]	June-Sep 2009	Before June 27 2009	Aug-Oct 2009	Sequential serological sampling from four cohorts: an existing community cohort, military conscripts, hospital staff, and long term care facility staff and residents.	Only the community cohort of persons aged 21-75 years (n = 838) was used in this review. The cohort was established to study aspects of chronic diseases.

a All studies used haemagglutination inhibition assays to assess infection unless indicated in the comments.

All studies included in the review included data on serum samples collected before and after the circulation of pH1N1, with some studies sampling at more than one time after the detection of pH1N1 circulation (Table 1). Circulation of pH1N1 occurred between May and November 2009 in all countries, independent of whether these countries were in the tropics or the temperate northern and southern hemispheres. Only in Norway was there no significant circulation of pH1N1 in any age group in the first wave during summer [61], while there appeared to be no significant circulation among adults in the study from England in the first wave and little significant circulation outside London prior to the second wave [4], [37].

The most common sampling strategy used convenience samples of residual diagnostic sera, often sourced from the laboratory in which the pandemic serosurvey was performed (Table 1). The studies from Canada [57], England [4], New South Wales [9], Western Australia [8], Norway [61] and the small study from Hong Kong [58] used residual diagnostic sera for both pre- and post-pandemic samples while the studies from India [59] and New Zealand [60] used residual diagnostic sera only for the pre-pandemic samples.

The study from Australia [55] used residual plasma from blood donors, while the large study from Hong Kong [10] included blood donors as only one of its sampling strategies. The large Hong Kong study also took advantage of an existing population-based cohort of children in addition to a sample of hospital outpatients. In Singapore existing cohorts of adults were sampled sequentially [62]. The study from India included a range of samples in its post-pandemic samples, including a survey of the ‘general population’ based on a cluster sample in 20 localities [59]. The study from Victoria used sequential serum sampling from an existing study based on a stratified random cluster sample of residents of the Melbourne metropolitan area [56]. Perhaps the most ambitious study design was the post-sampling strategy in the New Zealand study, which used a targeted cluster random sample of patients registered at sentinel general practices included in the influenza surveillance network in New Zealand [60].

In terms of the size of studies, the largest was the Hong Kong study [10] which included more than 15,000 serum samples, while the smallest was the study of pregnant women from Canada involving less than 600 serum samples [57]. Except for the large study from Hong Kong [10], which used microneutralisation assays, all other studies included in the review used haemagglutination inhibition assays.

### Age-specific infection

Estimates of pH1N1 infection in pre-school-age children (0-4 years) were reported only for New South Wales, Western Australia, England, India, Norway and New Zealand (Table 2). In Western Australia, New Zealand and regions of England that experienced a substantial first pandemic wave, reported estimates of the cumulative incidence of infection in pre-school aged children ranged from approximately 16%–28%. Lower estimates were found in Pune (India), Norway and some regions of England (Table 2). Estimates of the cumulative incidence of pH1N1 infection in school-aged children, broadly 5–19 years of age, were reported more frequently and were more similar around the world, with reported point estimates of the cumulative incidence in the range 34%–43% for most studies, although inconsistent results were reported on whether higher rates of infection were likely to occur in primary school-aged children (5–12 years) or older children. Where the age groups were separated, investigators from New South Wales and India reported higher infection rates in older children, while investigators from Hong Kong reported higher rates in younger children (Table 2).

**Table 2:** Reported age-specific cumulative incidence of infection for studies included in the review.

Study Location	Cumulative incidence (%) of infection by age groups (95% confidence interval [CI])			
	Pre-school aged children	School aged children	Adults	All ages
Australia [55]	Not reported	Not reported	10.0% (CI not reported), 16-78y	Not reported
New South Wales, Australia [9]	15.6% (9.9-21.4), <5y	9.8% (0.0-15.9), 5-11y 34.5% (24.0-44.7), 12-17y	8.8% (CI not reported), 18-≥85y	15.6% (estimate weighted by age and geographic region, CI not reported)
Victoria, Australia [56]	Not reported	Not reported	10.0% (CI not reported), 18-64y	Not reported
Western Australia [8]	25.4% (18.6-33.4), 1-4y	39.4% (29.8-48.5), 5-19y	10.2% (4.1-17.1), 21-45y (pregnant women only)	Not reported
Manitoba, Canada [57]	Not reported	Not reported	8.6% (3.2-13.7), 16-43y (pregnant women only)	Not reported
London & West Midlands, England [4] a	21.3% (8.8-40.3), <5y	42.0% (26.3-58.2), 5-14y	6.2% (-2.8-18.7), 25-44y -2.7% (-10.3-7.1), 45-64y 0.9% (-88.8-13.3), ≥65y	Not reported
Hong Kong [10]	Not reported	43.4% (37.9-47.6), 5-14y 15.8% (8.2-22.1), 15-19y	11.8% (8.4-14.7), 20-29y 4.3% (0.9-7.5), 30-39y 4.6% (1.0-7.9), 40-49y 4.0% (1.1-7.5), 50-59y	10.7% (9.0-12.0)
Hong Kong [58]	Not reported	23% (CI not reported), 10-19y	4% (CI not reported), 20-29y 9% (CI not reported), 30-39y 0% (CI not reported), 40-49y -14% (CI not reported), 50-65y -17% (CI not reported), >65y	Not reported
Pune, India [59]	7.4% (3.2-11.6), <5y	20.0% (17.2-22.7), 5-9y 26.7% (24.3-29.2), 10-14y 42.2% (36.1-48.3), 15-19y	6.0% (5.1-6.9), ≥20y (population) 10.7% (6.2-15.3) (school staff)	Not reported
New Zealand [60]	23.5% (CI not reported), 1-4y	32.7% (CI not reported), 5-19 y	14.7% (CI not reported), 20-39y 13.7% (CI not reported), 40-59y 2.2% (CI not reported), ≥60y	18.3% (age and ethnicity adjusted, CI not reported)
Norway [61]	0% (CI not reported), ≤2y	0% (CI not reported), 3-9y 4.9% (CI not reported), 10-19y	3.7% (CI not reported), 20-29y 0% (CI not reported), 30-49y 1.3% (CI not reported), 50-64y -1.6% (CI not reported), 65-79y 3.2% (CI not reported), >80y	1.5% (CI not reported)
Singapore [62]	Not reported	Not reported	13% (11-16), ≥20y	Not reported

a Only the region of London and the West Midlands is presented, as there was minimal pH1N1 circulation in other regions [4].

All included studies reported estimates of infection in adults. Again these were similar, in the range of approximately 9%–10%, where the reported age group ranged from 16–18 years up to 64 years, although no significant infection appeared to have occurred in adults in England during the first pandemic wave [4]. Where age groups were separated, all investigators reported higher infection rates in younger adults. New Zealand investigators reported approximately 15% of adults aged 20–39 years had been infected, whereas the estimate for this age group from the large Hong Kong study was approximately 12% (Table 2). Where it was measured, infection in adults older than 60 years was negligible, in the range 2%–3%. The reported estimates of infection in pregnant women were approximately 9% in Canada and 10% in Western Australia, not different to the estimates for adults of all ages, or for the estimates of similar aged adults in the large Hong Kong study (Table 2).

Because few studies reported estimates of infection in children, only the studies from Hong Kong, New South Wales and New Zealand reported estimates of infection for the entire population. The reported estimates were weighted by age and, in New Zealand, by ethnicity, to represent the population and ranged between approximately 11%–18% (Table 2).

### Re-calculated estimates of the cumulative incidence of infection

**Figure 2:**
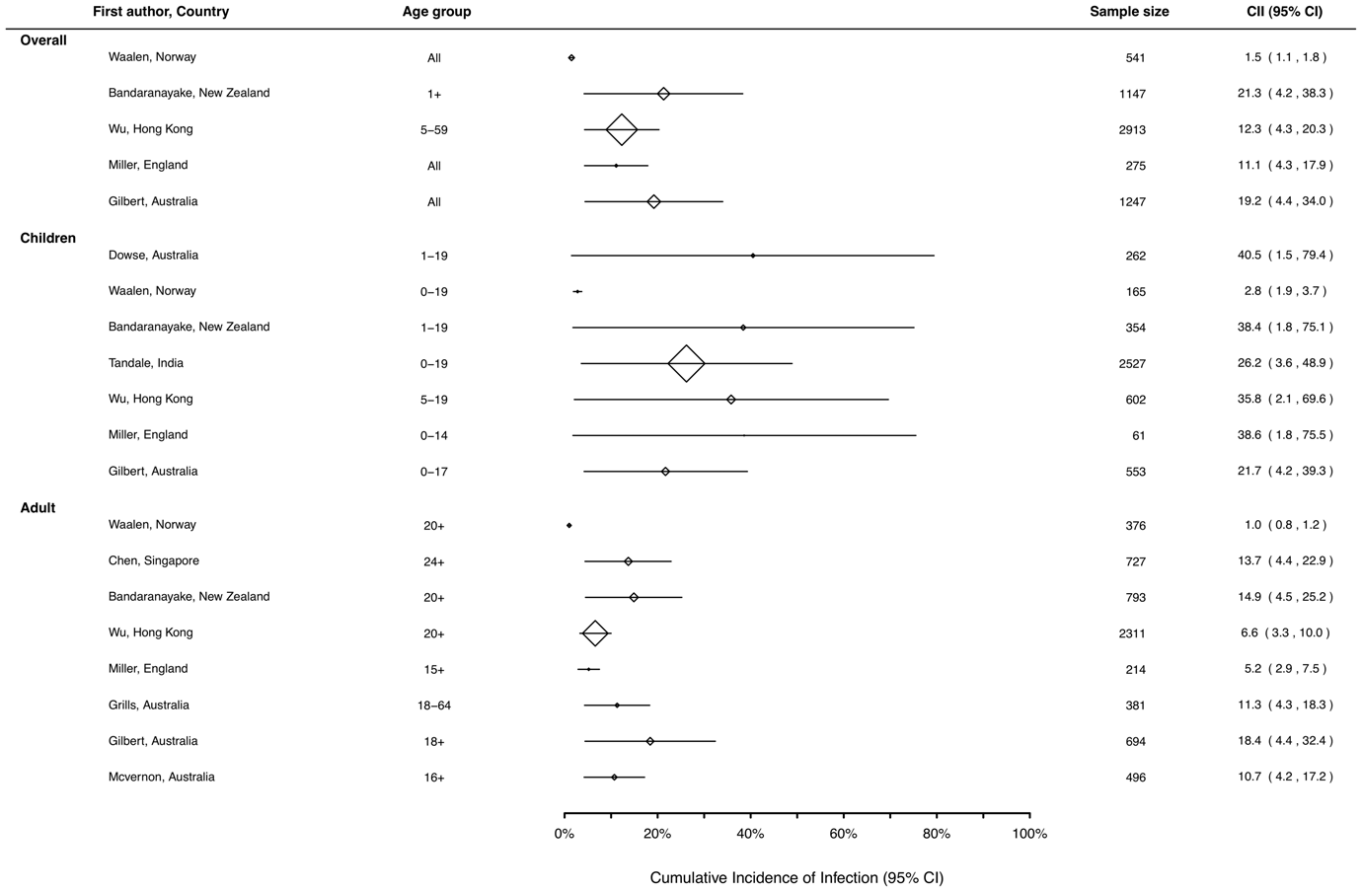
Cumulative Incidence of Infection (95% CI).

As described in the methods we re-calculated the cumulative incidence of infection, where this was possible from the data provided in the original study, inflating the final sero-prevalence estimate by 10% to account for infections that may have been missed by serology, and performing direct age-standardisation of the estimates for each country or state. This resulted in some minor changes to the estimates of the cumulative incidence of infection, such as a slightly higher estimate of infection in the New Zealand population (18% reported compared to 21% re-calculated) and New South Wales (16% reported compared with 19% re-calculated). Most other re-calculated estimates were similar to the reported estimates (Table 3). However when we re-estimated 95% confidence intervals for the re-calculated cumulative incidence of infection for children, adults and all ages, we found that the smaller studies had substantial uncertainty about the re-estimated cumulative incidence of infection (Figure 2).

### Supporting findings from excluded studies

**Table 3:** Estimated age-standardized cumulative incidence of pandemic H1N1 influenza infection in ten studiesa.

Study Location	Age group (years)	Cumulative incidence of infection (%) (95% CI)
Australia [55]	≥16	10.7 (4.2, 17.2)
New South Wales, Australia [9]	All	19.2 (4.4, 34.0)
Victoria, Australia [56]	18-64	11.3 (4.3, 18.3)
Western Australia [8]	1-19	40.5 (1.5, 79.4)
England^b^ [4]	All	11.1 (4.3, 17.9)
Hong Kong [10]	5-59	12.3 (4.3, 20.3)
India [59]	0-19	26.2 (3.6, 48.9)
New Zealand [60]	≥1	21.3 (4.2, 38.3)
Singapore [62]	≥24	13.7 (4.4, 24.9)
Norway [61]	All	1.5 (1.1, 1.8)

a Two of 12 studies were excluded due to the selective sample (only pregnant women) in the Canadian study [57] and the small sample size in one Hong Kong study [58].

b Age-standardized cumulative incidence in the region of London and the West Midlands was used, as there was minimal pH1N1 circulation in other regions [4].

We further reviewed formally excluded studies to see if any of them provided support for our findings. Where there was evidence of low pre-pandemic seroprevalence from a study in a similar population base when a study reported only post-pandemic samples, we assumed that the related pre-pandemic studies might apply to the post-pandemic estimates in that population. When there were only post-pandemic results for children, we assumed that pre-pandemic titres may have been effectively zero, given this was found in some of the included studies. Although we excluded six studies from China, four of these studies provided potentially useful estimates of infection (Table S1). Prior to circulation of pH1N1, only about 2% of the resident population of Guangxi province of China had cross-reacting pH1N1 antibodies [32]. This study had been excluded because it only estimated seroprevalence prior to the circulation of pH1N1. However if this pre-pandemic seroprevalence were true for all of China, two serosurveys from Beijing that only estimated infection post pH1N1 circulation, might provide representative estimates of infection in China. An age-weighted estimate of the cumulative incidence of infection in the Beijing population was reported as 14% [33], with estimates of 25% in children 0–5 years and 34% in children 6–15 years from another Beijing study [35]. These estimates are consistent with both the age-specific and population estimates of pH1N1 infection from the studies included in the review. A study of 200 blood donors aged 19–55 years from Guangzhou, China [36], excluded because of the absence of pre-pandemic testing, reported a post-pandemic sero-prevalence of 11%. This would be consistent with other studies only if the pre-pandemic sero-prevalence in these blood donors was effectively zero.

In another brief report from Beijing, excluded from this review because of no pre-pandemic testing [31], the seroprevalence in persons (age not specified) who had not received a pandemic vaccine increased from approximately 14% to 28% between late December 2009/early January 2010 and March 2010.

In a study from the US, excluded because of unequal pre- and post-samples structures [52], it is possible to use the post-pandemic estimates to assess the cumulative incidence of infection in children if we assume that pre-pandemic titres were zero. Collected 2–4 weeks after the second wave from 846 persons aged 1 month to 90 years, serum samples indicated 28% of children aged 0–9 years were sero-positive for pH1N1 and 45% of the 10–19 years age group were reported as seropositive. The numbers tested in each age group were not reported and it was not possible to re-calculate confidence intervals. Nonetheless these point estimates of the cumulative incidence of infection in these age groups are consistent with those from the included studies.

## Discussion

From a search for serosurveys published within a year of the end of the first pandemic wave in the northern hemisphere and the end of the season in which pH1N1 predominated in the southern hemisphere, we found 12 studies that estimated the cumulative incidence of pH1N1 infection. A further five studies, excluded because they did not provide both pre- and post-pandemic estimates, provided supportive estimates of the cumulative incidence of infection. The exclusion of the majority of original non-duplicate studies identified by our search illustrates the problems of conducting adequate sero-surveys on short notice, especially when sero-surveys were not planned as an element of pandemic evaluation. We needed to adopt relaxed criteria on the assessment of sampling strategies in order to include the final 12 studies. In particular no sample was random, although the routinely collected serum samples from Norway were representative of age and region [61]; there were only two cohort studies - in Hong Kong [10] and Singapore [62]; pre- and post-pandemic sampling was usually unbalanced, with more extensive sampling post-pandemic; and timing of the post-pandemic samples was not always optimal, sometimes being prior to the end of pH1N1 circulation [8]. The increase in the reported seroprevalence of an antibody titre >40 amongst unvaccinated persons in Beijing between December 2009 and March 2010 illustrates the importance of sample timing [31].

Moreover even the use of standard serological assays apparently did not guarantee detection of all infected persons. Ten percent of 881 patients from Hong Kong confirmed to have pH1N1 infection by PCR testing had a neutralising antibody titre <40 [12] and would have been below the conventional cut-off used to determine seroprotection. In this study being afebrile at presentation was associated with a lower titre (p = 0.04), suggesting that some afebrile or completely asymptomatic infections may have been missed – even in the serosurveys. Use of antiviral treatment was associated with low convalescent antibody titres [63] further potentially affecting the results of serologic studies in countries which made substantial use of antiviral treatment. We attempted to adjust for these findings in re-calculating estimates of the cumulative incidence of infection. We also re-calculated confidence intervals, based on the assumption of simple random sampling. These confidence intervals provide an indication of the relative uncertainty of the point estimates of the cumulative incidence from the included studies.

### Findings from this study

Given these important reservations, we found that, with the exception of pre-school aged children, the reported age-specific estimates of infection with pH1N1 were similar in all studies where there had been a significant first wave of infection, in the range of 34%–43% for school-aged children and around 10% for adults, but much lower for adults aged at least 60 years. Where the cumulative incidence of infection could be estimated for the population of all ages, it was reported to range from 11% in Hong Kong [10] to 18% in New Zealand [60], with an intermediate estimate of 16% in New South Wales [9]. A study from Beijing, excluded because of the absence of pre-pandemic sampling, reported infection in people aged 0–≥60 years of 14% [33]. Re-calculation of the cumulative incidence of infection in the whole population resulted in slightly higher estimates in the range 11% (4%–18%) in England to 21% (4%–38%) in New Zealand.

A study from Iran reported post-pandemic titres >40 in 50–60% of children aged from 0–19 years [41]. No pre-pandemic testing was performed and this study was excluded from the review. If the proportion of pre-pandemic titres >40 was effectively zero in Iran, as it appears to have been in many other countries where testing of children was done, the cumulative incidence of infection in Iran would be the highest of all reported countries. Of interest is that all age groups in this study were recorded as having 50–60% of tested persons with a pH1N1 titre >40.

The pandemic in the southern hemisphere, occurring during the expected influenza season, probably resulted in more uniform infection throughout the population than did the first and second pandemic waves in the northern hemisphere, which were characterised by variable infection rates between countries, such as England [4] and Norway [61] and within countries [4], [37], [43]. Nonetheless this review confirms that cumulative incidence of infection during the first and second waves prior to the availability of a pandemic specific vaccine anywhere in the world, fell well below the assumptions of an upper estimate of a 50% clinical attack rate that had informed the pandemic planning of the United Kingdom and other countries [64], [65], [66].

The lower than anticipated cumulative incidence was likely due to the significant number of older people protected by neutralising antibodies that cross-reacted with pH1N1 [2], [38], [42], [50]. Indeed from the serosurveys it appears as if infection was not common, even in adults aged >40 years. Effectively a small proportion of infection in a large proportion of the population resulted in lower than expected estimates for the cumulative incidence of infection in the whole population. The review also confirms that school-aged children were most frequently infected with pH1N1, presumably reflecting mixing patterns and relative immunological naivety in this age group.

### Study design and sample size

The studies ranged in size from many thousands of serum samples, such as those in India [59] and Hong Kong [10], to hundreds of serum samples, such as the study of pregnant women in Canada [57]. Convenience sampling of residual diagnostic sera was the most common single approach to sampling in these studies. Residual samples are used extensively throughout the world to evaluate immunisation programs [61], [67], and one previous study has suggested that residual diagnostic samples gave similar estimates of immunity to a range of vaccine preventable diseases as were obtained by a random cluster sample [68]. While residual diagnostic samples are limited by potential biases relating to risk of infection and vaccination history in the predominantly inpatient population from which they are typically drawn, similar estimates of the cumulative incidence of infection were obtained using sera from healthy blood donors.

Studies in Hong Kong [10] and Singapore [62] were able to take advantage of existing cohorts. Results from the cohort study in Hong Kong were similar to the results from the cross-sectional surveys in that city and the results from the adult cohort in Singapore were similar to results for adults from a range of other sampling strategies around the world. Study design and sample size may not have been as critical as theoretical considerations would have suggested, although the larger samples resulted in much better precision of the point estimates, as shown by our re-estimates of the confidence intervals. It has been suggested post-pandemic sero-prevalence surveys need to test thousands of specimens for their results to be informative. [20]

A somewhat surprising result from the review was the similarity of point estimates of cumulative incidence from large well designed studies to those obtained from smaller studies using residual sera. The ideal study design would recruit a longitudinal cohort of randomly selected people of all ages. Comparison of results from this review with anticipated ideal design studies will provide a more definitive answer to the question of whether samples of residual diagnostic sera are adequate to determine evidence-based policy for influenza vaccination.

### Implications from our findings

Compared to seasonal influenza [69], a higher proportion of asymptomatic infection for pH1N1 has been suggested [22]. Moreover some studies suggested a substantial proportion of infected people did not present for medical attention [4], [60]. Using results from their serosurveys and including information from other studies, investigators have suggested the case fatality ratio (CFR) for pH1N1 may have varied between 0.01% [55] and 0.008% [60]. Although these CFR estimates are probably based on under-estimates of death due to pH1N1, they are two orders of magnitude lower than estimates of the CFR up to 2% that have been widely accepted for the pandemic of 1918–19.

If serological studies are to be sufficiently timely to inform policy, they need to be collected routinely, as is done in Norway [61] and the UK [4]. However serological studies are most informative towards the end of the pandemic. Other methods for estimating cumulative infection of pH1N1 infection earlier in the pandemic were based on existing surveillance systems. Comparisons of estimates from these systems with those from serological studies in Singapore showed that modelled estimates of the cumulative incidence of pH1N1 infection from laboratory-supported sentinel surveillance schemes were similar to those from serological surveys [70], suggesting that modelling may have an important role in the early estimates of incidence and cumulative incidence.

In conclusion we found that serological studies that estimated the cumulative incidence of pH1N1 infection around the world confirmed the findings from surveillance that school-aged children were the most commonly infected age group and older adults were relatively spared. Serological studies need to be routine in order to be sufficiently timely to provide support for decisions about vaccination and revised pandemic plans might consider a more integrated role for serological studies.

## Supporting Information

Table S1 **Twenty-nine studies excluded from the review and reasons for exclusion.**
(DOC)

## References

[1] World Health Organization website, Chan M. (2009). World now at the start of an influenza pandemic.. http://wwwwhoint/mediacentre/news/statements/2009/h1n1_pandemic_phase6_20090611/en/indexhtml.

[2] Hancock K, Veguilla V, Lu X, Zhong W, Butler EN (2009). Cross-reactive antibody responses to the 2009 pandemic H1N1 influenza virus.. N Engl J Med.

[3] Baker M, Kelly H, Wilson N (2009). Pandemic H1N1 influenza lessons from the southern hemisphere..

[4] Miller E, Hoschler K, Hardelid P, Stanford E, Andrews N (2010). Incidence of 2009 pandemic influenza A H1N1 infection in England: a cross-sectional serological study.. Lancet.

[5] Last JM (2001). A Dictionary of Epidemiology, 4th Edition..

[6] Reed C, Angulo FJ, Swerdlow DL, Lipsitch M, Meltzer MI (2009). Estimates of the prevalence of pandemic (H1N1) 2009, United States, April–July 2009.. Emerg Infect Dis.

[7] Baker MG, Wilson N, Huang QS, Paine S, Lopez L (2009). Pandemic influenza A(H1N1)v in New Zealand: the experience from April to August 2009..

[8] Dowse GK, Smith DW, Kelly H, Barr I, Laurie KL (2011). Incidence of pandemic (H1N1) 2009 influenza infection in children and pregnant women during the 2009 influenza season in Western Australia - a seroprevalence study.. Med J Aust.

[9] Gilbert GL, Cretikos MA, Hueston L, Doukas G, O'Toole B (2010). Influenza A (H1N1) 2009 antibodies in residents of New South Wales, Australia, after the first pandemic wave in the 2009 southern hemisphere winter.. PLoS One.

[10] Wu JT, Ma ES, Lee CK, Chu DK, Ho PL (2010). The infection attack rate and severity of 2009 pandemic H1N1 influenza in Hong Kong.. Clin Infect Dis.

[11] Gurav YK, Pawar SD, Chadha MS, Potdar VA, Deshpande AS (2010). Pandemic influenza A(H1N1) 2009 outbreak in a residential school at Panchgani, Maharashtra, India.. Indian J Med Res.

[12] Hung IF, To KK, Lee CK, Lin CK, Chan JF (2010). Effect of clinical and virological parameters on the level of neutralizing antibody against pandemic influenza A virus H1N1 2009.. Clin Infect Dis.

[13] Australian Bureau of Statistics website (2010). Australian Demographic Statistics, Sep 2010. Data Cubes: Population by Age and Sex.. http://www.abs.gov.au/AUSSTATS/abs@.nsf/DetailsPage/3101.0Sep%202010?OpenDocument.

[14] Office for National Statistics UK website (2011). Vital Statistics: Population and Health Reference Tables. 1.4: Population: age and sex, constituent countries of the UK.. http://wwwstatisticsgovuk/StatBase/Productasp?vlnk=15354.

[15] Census and Statistics Department: Hong Kong website (2011). Statistical Tables: Table 002: Population by Age Group and Sex.. http://wwwcenstatdgovhk/hong_kong_statistics/statistical_tables/indexjsp?charsetID=1&tableID=002.

[16] Ministry of Health & Family Welfare, India website (2009). Section A - Population and Vital Statistics.. http://nrhm-misnicin/familywelfare2009html.

[17] Statistics New Zealand website (2010). National Population Estimates: June 2010 quarter – Tables.. http://wwwstatsgovtnz/browse_for_stats/population/estimates_and_projections/NationalPopulationEstimates_HOTPJun10qtraspx.

[18] Department of Statistics: Singapore website (2010). Yearbook of Statistics Singapore 2010: 3.4 Singapore Residents by Age Group, Ethnic Group and Sex, End June 2009.. http://wwwsingstatgovsg/pubn/reference/yos10/yos2010pdf.

[19] Statistics Norway website (2010). Population, by age and county. Absolute figures. 1 January 2010.. http://wwwssbno/folkemengde_en/arkiv/tab-2010-02-18-01-enhtml.

[20] Nishiura H, Chowell G, Castillo-Chavez C (2011). Did modeling overestimate the transmission potential of pandemic (H1N1-2009)? Sample size estimation for post-epidemic seroepidemiological studies.. PLoS One.

[21] Rogan WJ, Gladen B (1978). Estimating prevalence from the results of a screening test.. Am J Epidemiol.

[22] Flahault A, de Lamballerie X, Hanslik T (2009). Symptomatic infections less frequent with H1N1pdm than with seasonal strains.. PLoS Curr Infl.

[23] Itoh Y, Shinya K, Kiso M, Watanabe T, Sakoda Y (2009). In vitro and in vivo characterization of new swine-origin H1N1 influenza viruses.. Nature.

[24] Reed C, Katz JM (2010). Serological surveys for 2009 pandemic influenza A H1N1.. Lancet.

[25] Liu W, Yang P, Duan W, Wang X, Zhang Y (2010). Factors associated with seropositivity of 2009 H1N1 influenza in Beijing, China.. Clin Infect Dis.

[26] Wiwanitkit V (2010). 2009 H1N1 influenza virus seroepidemiology.. Iran J Immunol 7: 130; author reply.

[27] World Health Organization. (2010). Seroepidemiological studies of pandemic influenza A (H1N1) 2009 virus.. Wkly Epidemiol Rec.

[28] Ross T, Zimmer S, Burke D, Crevar C, Carter D (2010). Seroprevalence Following the Second Wave of Pandemic 2009 H1N1 Influenza.. PLoS Curr.

[29] Katz J, Hancock K, Veguilla V, Zhong W, Lu XH (2009). Serum cross-reactive antibody response to a novel influenza A (H1N1) virus after vaccination with seasonal influenza vaccine.. MMWR Morb Mortal Wkly Rep.

[30] Skowronski DM, Hottes TS, Janjua NZ, Purych D, Sabaiduc S (2010). Prevalence of seroprotection against the pandemic (H1N1) virus after the 2009 pandemic.. CMAJ.

[31] Yang P, Shi W, Tian L, Li S, Zhang L (2011). Serological surveillance of 2009 H1N1 influenza in China.. Int J Infect Dis.

[32] Chen H, Wang Y, Liu W, Zhang J, Dong B (2009). Serologic survey of pandemic (H1N1) 2009 virus, Guangxi Province, China.. Emerg Infect Dis.

[33] Deng Y, Pang XH, Yang P, Shi WX, Tian LL (2011). Serological survey of 2009 H1N1 influenza in residents of Beijing, China.. Epidemiol Infect.

[34] Jiang T, Li X, Liu W, Yu M, Liu J (2010). Serum Antibody Response to the Novel Influenza A (H1N1) Virus in the Elderly.. Clin Infect Dis.

[35] Tian LL, Shi WX, Ying D, Pang XH, Peng Y (2010). Serologic survey of pandemic influenza A (H1N1 2009) in Beijing, China.. Prev Med.

[36] Zhang R, Rong X, Pan W, Peng T (2011). Determination of serum neutralization antibodies against seasonal influenza A strain H3N2 and the emerging strains 2009 H1N1 and avian H5N1.. Scand J Infect Dis.

[37] Hardelid P, Andrews NJ, Hoschler K, Stanford E, Baguelin M (2010). Assessment of baseline age-specific antibody prevalence and incidence of infection to novel influenza A/H1N1 2009.. Health Technol Assess.

[38] Ikonen N, Strengell M, Kinnunen L, Osterlund P, Pirhonen J (2010). High frequency of cross-reacting antibodies against 2009 pandemic influenza A(H1N1) virus among the elderly in Finland..

[39] Aho M, Lyytikainen O, Nyholm JE, Kuitunen T, Ronkko E (2010). Outbreak of 2009 pandemic influenza A(H1N1) in a Finnish garrison--a serological survey..

[40] Allwinn R, Geiler J, Berger A, Cinatl J, Doerr HW (2010). Determination of serum antibodies against swine-origin influenza A virus H1N1/09 by immunofluorescence, haemagglutination inhibition, and by neutralization tests: how is the prevalence rate of protecting antibodies in humans?. Med Microbiol Immunol.

[41] Moghadami M, Moattari A, Tabatabaee HR, Mirahmadizadeh A, Rezaianzadeh A (2010). High titers of hemagglutination inhibition antibodies against 2009 H1N1 influenza virus in Southern Iran.. Iran J Immunol.

[42] Rizzo C, Rota MC, Bella A, Alfonsi V, Declich S (2010). Cross-reactive antibody responses to the 2009 A/H1N1v influenza virus in the Italian population in the pre-pandemic period.. Vaccine.

[43] Adamson WE, Maddi S, Robertson C, McDonagh S, Molyneaux PJ (2010). 2009 pandemic influenza A(H1N1) virus in Scotland: geographically variable immunity in Spring 2010, following the winter outbreak..

[44] Chen MI, Barr IG, Koh GC, Lee VJ, Lee CP (2010). Serological response in RT-PCR confirmed H1N1-2009 influenza A by hemagglutination inhibition and virus neutralization assays: an observational study.. PLoS One.

[45] Lee VJ, Yap J, Tay JK, Barr I, Gao Q (2010). Seroconversion and asymptomatic infections during oseltamivir prophylaxis against Influenza A H1N1 2009.. BMC Infect Dis.

[46] Lee VJ, Yap J, Cook AR, Chen MI, Tay JK (2010). Effectiveness of public health measures in mitigating pandemic influenza spread: a prospective sero-epidemiological cohort study.. J Infect Dis.

[47] Chen MI, Lee VJ, Barr I, Lin C, Goh R (2010). Risk factors for pandemic (H1N1) 2009 virus seroconversion among hospital staff, Singapore.. Emerg Infect Dis.

[48] Tang JW, Tambyah PA, Wilder-Smith A, Puong KY, Shaw R (2010). Cross-reactive antibodies to pandemic (H1N1) 2009 virus, Singapore.. Emerg Infect Dis.

[49] Chan YJ, Lee CL, Hwang SJ, Fung CP, Wang FD (2010). Seroprevalence of antibodies to pandemic (H1N1) 2009 influenza virus among hospital staff in a medical center in Taiwan.. J Chin Med Assoc.

[50] Chang SC, Chang CM, Huang YC, Chiu CH, Shih SR (2010). Preexisting antibodies against pandemic 2009 influenza A (H1N1) virus in Taiwan.. Clin Infect Dis.

[51] Chi CY, Liu CC, Lin CC, Wang HC, Cheng YT (2010). Preexisting antibody response against 2009 pandemic influenza H1N1 viruses in the Taiwanese population.. Clin Vaccine Immunol.

[52] Zimmer SM, Crevar CJ, Carter DM, Stark JH, Giles BM (2010). Seroprevalence following the second wave of Pandemic 2009 H1N1 influenza in Pittsburgh, PA, USA.. PLoS One.

[53] Tsai TF, Pedotti P, Hilbert A, Lindert K, Hohenboken M (2010). Regional and age-specific patterns of pandemic H1N1 influenza virus seroprevalence inferred from vaccine clinical trials, August-October 2009..

[54] Greenbaum JA, Kotturi MF, Kim Y, Oseroff C, Vaughan K (2009). Pre-existing immunity against swine-origin H1N1 influenza viruses in the general human population..

[55] McVernon J, Laurie K, Nolan T, Owen R, Irving D (2010). Seroprevalence of 2009 pandemic influenza A(H1N1) virus in Australian blood donors, October - December 2009..

[56] Grills N, Piers LS, Barr I, Vaughan LM, Lester R (2010). A lower than expected adult Victorian community attack rate for pandemic (H1N1) 2009.. Aust N Z J Public Health.

[57] Mahmud SM, Becker M, Keynan Y, Elliott L, Thompson LH (2010). Estimated cumulative incidence of pandemic (H1N1) influenza among pregnant women during the first wave of the 2009 pandemic.. CMAJ.

[58] Mak GC, Choy PW, Lee WY, Wong AH, Ng KC (2010). Sero-immunity and serologic response to pandemic influenza A (H1N1) 2009 virus in Hong Kong.. J Med Virol.

[59] Tandale BV, Pawar SD, Gurav YK, Chadha MS, Koratkar SS (2010). Seroepidemiology of pandemic influenza A (H1N1) 2009 virus infections in Pune, India.. BMC Infect Dis.

[60] Bandaranayake D, Huang QS, Bissielo A, Wood T, Mackereth G (2010). Risk factors and immunity in a nationally representative population following the 2009 influenza A(H1N1) pandemic.. PLoS One.

[61] Waalen K, Kilander A, Dudman SG, Krogh GH, Aune T (2010). High prevalence of antibodies to the 2009 pandemic influenza A(H1N1) virus in the Norwegian population following a major epidemic and a large vaccination campaign in autumn 2009.. Euro Surveill 15: pii =.

[62] Chen MI, Lee VJ, Lim WY, Barr IG, Lin RT (2010). 2009 influenza A(H1N1) seroconversion rates and risk factors among distinct adult cohorts in Singapore.. JAMA.

[63] Cowling BJ, Chan KH, Fang VJ, Lau LL, So HC (2010). Comparative epidemiology of pandemic and seasonal influenza A in households.. N Engl J Med.

[64] Department of Health website website (2007). Pandemic flu: a national framework for responding to an influenza pandemic. London: Department of Health, United Kingdom. 141 p.. http://www.hpa.org.uk/web/HPAwebFile/HPAweb_C/1238055320501.

[65] Australian Government Department of Health and Ageing website (2008). Australian Health Management Plan for Pandemic Influenza. Canberra: Australian Government Department of Health and Ageing.. http://www.flupandemic.gov.au/internet/panflu/publishing.nsf/Content/ahmppi-2009.

[66] World Health Organization website (2009). Global Influenza Program: Pandemic Influenza Preparedness and Response. Geneva: World Health Organization.. http://www.who.int/csr/disease/influenza/pipguidance2009/en/index.html.

[67] Health Protection Agency UK webiste (2010). Seroepidemiology programme: sample collection.. http://www.hpa.org.uk/ProductsServices/InfectiousDiseases/ServicesActivities/SeroepidemiologyProgramme/.

[68] Kelly H, Riddell MA, Gidding HF, Nolan T, Gilbert GL (2002). A random cluster survey and a convenience sample give comparable estimates of immunity to vaccine preventable diseases in children of school age in Victoria, Australia.. Vaccine.

[69] Carrat F, Vergu E, Ferguson NM, Lemaitre M, Cauchemez S (2008). Time lines of infection and disease in human influenza: a review of volunteer challenge studies.. Am J Epidemiol.

[70] Lee VJ, Chen MI, Yap J, Ong J, Lim W (2011). Comparability of different methods for estimating influenza infection rates over a single epidemic wave..

