# Supplementary material for: The Age-Specific Cumulative Incidence of Infection with Pandemic Influenza H1N1 2009 Was Similar in Various Countries Prior to Vaccination
Source: PLoS One. 2011 Aug 5;6(8):e21828. doi: 10.1371/journal.pone.0021828 (PMC3151238; doi:10.1371/journal.pone.0021828)
Supplement: Table S1 — Twenty-nine studies excluded from the review and reasons for exclusion. (DOC) [file pone.0021828.s001.doc]

Table S1: Twenty-eight studies excluded from the review and reasons for exclusion

| Country (reference) | Study design | Reasons for study exclusion |
| --- | --- | --- |
| Canada [30] | Residual serum samples collected from patients aged <5 to >90 years attending one of a number of community laboratory centres across British Columbia (n=1127) | Not able to distinguish antibody response from natural infection with that from vaccination |
| Guangxi province, China [32] | 4043 stored serum samples collected from residents of the province, aged 7-84 years, in 2008 | The study aimed to determine antibodies to pH1N1 prior to the circulation of the virus. Seroprevalence was ~ 2%. There was no post-pandemic sample. |
| Beijing, China [33] | Serological survey of Beijing residents: 710 serum samples from blood donors and hospital outpatients, aged 0- >60 years. | The study determined the seroprevalence of antibodies to pH1N1 one month after the epidemic peak. There was no pre-pandemic sample and 35 participants had received pH1N1 vaccine. Age-weighted antibody prevalence was 14%. |
| Beijing, China [34] | Serological study of elderly residents in Beijing, China. Pre-pandemic sample collected in April 2009 (n=32), post-pandemic sample collected in September 2009 (n=80). Age range 60-87 years. | Not able to distinguish antibody response from natural infection with that from vaccination in the post-pandemic sample. Pre-pandemic antibody prevalence 9.4%, post-pandemic prevalence 42.5%. |
| Beijing, China [35] | Multi-stage stratified sampling from six randomly selected geographic districts (four city districts and two country districts): 4601 subjects participated. | No pre-pandemic testing undertaken. 1336 subjects received pH1N1 vaccine, with a seropositivity rate of ~ 61%. 3265 subjects with no pH1N1 vaccine history had an overall seropositivity rate of 20%, with children 0-5 years 25% and children 6-15 years 34% seropositive. |
| Beijing, China [31] | Serological surveillance of 2009 H1N1 influenza conducted over three time periods: (1) December 31 2009 – January 8 2010, n=329 (2) January 20 2010 – January 28 2010, n=470 and (3) March 9 2010 – March 20 2010, n=400. | No pre-pandemic testing undertaken. Age-weighted seroprevalence for time period (1): 14.4%, time period (2): 17.7%, time period (3): 29.7% |
| Guangzhou, China [36] | Serological survey of 200 blood donors aged 19-55 years in Guangzhou, collected in October 2009. | No pre-pandemic testing undertaken. Post pandemic prevalence 11%. |
| England [37] | Study of the first and second waves in England up until April 2010, using residual sera from diagnostic and reference laboratories. | Although pre-pandemic estimates of seroprevalence were made, post-pandemic estimates after November 2009, when the pandemic vaccine became available, could not distinguish an antibody response to infection from a response due to vaccination. |
| Finland [38] | Residual serum samples from a viral diagnostic laboratory, collected in 2004 and 2005 (n=1031). | Study of cross-reacting antibodies prior to the circulation of pH1N1 |
| Finland [39] | Outbreak investigation in a garrison of soldiers, 346 sera tested from 984 recruits | pH1N1 antibodies were detected in 49% of soldiers. There was no pre-pandemic sample and the setting was not representative. |
| France [22] | Seroprevalence survey among a sample of pregnant women | No pre-pandemic sample. The pH1N1 antibody prevalence was ~11%. |
| Germany [40] | Pre: 145 sera from healthy blood donors, 13% with cross-reacting antibodies. Post: 225 sera from hospital inpatients and outpatients, 12% with pH1N1 antibodies | Post pandemic sample only from hospital patients, not representative of the community. |
| Hong Kong [12] | Study of 881 convalescent donors from case patients presenting with influenza-like illness, both hospitalised and outpatients. | The study reported the serological response following confirmed pH1N1 infection. |
| India [11] | Outbreak analysis of an entire school population (n=415). | No pre-pandemic testing undertaken. HI antibody titre of 10 used as a positive antibody response. The reported seropositivity in school children of 52% may have reflected the lower threshold for the HI assay. |
| Iran [41] | Random cluster sample of 2553 residents from Southern Iran | No pre-pandemic sample. Antibody prevalence reported as ~59%, highest in age group 60-64 years. |
| Italy [42] | National cross-sectional seroprevalence study (n=587) of residual diagnostic sera obtained from people aged <1 to >65 years collected in 2003 and 2004. | Study of cross-reacting antibodies prior to the circulation of pH1N1 |
| Japan [23] | Study of two donor groups from a broad age-range: (1) collected from residents and workers in nursing home, 1999 (n=64) and (2) from workers and patients in a hospital, April 2009 (n=214) | Study of cross-reacting antibodies prior to the circulation of pH1N1. |
| Scotland [43] | 1600 residual sera obtained from hospitals/general practices in four geographical areas and four age groups (100 sera per area and age group). 100 residual sera taken from each of four specialised clinics and an additional 127 residual sera taken from hospital outpatients over 50 years of age. | No pre-pandemic testing undertaken. Unable to distinguish between antibodies from natural infection and/or vaccination. |
| Singapore [44] | Observational study of three groups: hospitalised cases(n=67), sero-incidence cohort (n=28) and outbreak cases (n=25). | The study reported the serological response following confirmed pH1N1 infection. |
| Singapore [45] | Sera from three outbreaks in military cohorts, including 237 persons, tested at three time points. | Post-exposure oseltamivir prophylaxis trial in an outbreak setting. The study was not community-based. |
| Singapore [46] | Sera from three prospective military cohorts: normal personnel (n=472), essential personnel (n=567) and health care workers (n=164), tested at three time points. | Investigation of effectiveness of public health measures in an outbreak setting. The study was not community-based. |
| Singapore [48] | Sera from 50 randomly recruited, healthy volunteers sampled between May-June 2009, prior to widespread transmission of pH1N1 in Singapore. | Study of cross-reacting antibodies prior to the circulation of pH1N1 |
| Singapore [47] | Cohort of healthcare workers forming part of a larger seroepidemiologic study included in this review [62], n=558. | Sample only from healthcare workers, not representative of the community. |
| Taiwan [49] | 295 serum samples from hospital staff and 244 serum samples from outpatients, both collected in the post-pandemic period. | No pre-pandemic sample. Antibody prevalence in hospital staff with mean age 34-39 years varied from 13%-20% while antibody prevalence in control group with a median age of 52 years was 3%. |
| Taiwan [50] | 37% of 79 persons aged > 60 years from Taoyuan County, collected February-March 2009 (pre-pandemic), had cross-reacting antibodies. 8% of 169 healthcare workers aged 22-60 years had pH1N1 antibodies after the first pandemic wave. | Study of cross-reacting antibodies in the elderly prior to the circulation of pH1N1 and antibody-prevalence after circulation of pH1N1 in health care workers. No pre-pandemic sample in health care workers and no post-pandemic sample in the elderly. |
| Taiwan [51] | 229 serum samples from donors born between 1917 and 2008, collected in 2007 and 2008. | Study of cross-reacting antibodies prior to the circulation of pH1N1. 59% of subjects over the age of 79 years had a 2009 pH1N1 titre > 160. |
| USA[2,29] | Stored serum panels from vaccine trials and blood donors collected in 1976 and 2005-2009 were sourced from academic, government and industry partners. | Study of cross-reacting antibodies prior to the circulation of pH1N1 |
| USA [28,52] | Pre: sera from healthy adults aged 18-24 years, sampled in 2008. Post: excess sera from individuals aged 1 month to 90 years, collected November-December 2009. | Pre-pandemic sera samples (n=100) were sourced from young healthy adults while post-pandemic samples (n=846) were sourced from children and adults of all ages. Unable to determine age-specific infection from this study design. |
| USA, Germany, Belgium, Switzerland, Costa Rica, Japan [53] | Pre-vaccination clinical trial sera from 7,962 individuals across the USA, Costa Rica, Europe and Japan. Age range >1 year to >60 years, collected between August and October 2009. | No pre-pandemic testing undertaken. Antibody prevalence ranged between 26.4% (Costa Rica) and 5.9% (Japan) |
